# Strategies to support culturally safe health and wellbeing evaluations in Indigenous settings in Australia and New Zealand: a concept mapping study

**DOI:** 10.1186/s12939-019-1094-z

**Published:** 2019-12-16

**Authors:** Margaret Cargo, Gill Potaka-Osborne, Lynley Cvitanovic, Lisa Warner, Sharon Clarke, Jenni Judd, Amal Chakraborty, Amohia Boulton

**Affiliations:** 1 Health Research Institute, University of Canberra, Canberra, Australia; 2 School of Health Sciences, University of South Australia, Adelaide, Australia; 3 Whakauae Research for Māori Health and Development, Whanganui, New Zealand; 4 Aboriginal Women’s Leadership Program, Young Women Christian Association Australia, Adelaide, Australia; 5 South Australian Department of Health and Wellbeing, Adelaide, Australia; 6 Centre for Indigenous Health Equity Research, School of Health Medical and Applied Sciences, Central Queensland University Australia, Bundaberg, Australia

**Keywords:** Evaluation, Indigenous, Māori, Aboriginal and Torres Strait islander, Cultural safety, Concept mapping, Commissioning, Health promotion

## Abstract

**Background:**

In recent decades, financial investment has been made in health-related programs and services to overcome inequities and improve Indigenous people’s wellbeing in Australia and New Zealand. Despite policies aiming to ‘close the gap’, limited evaluation evidence has informed evidence-based policy and practice. Indigenous leaders have called for evaluation stakeholders to align their practices with Indigenous approaches.

**Methods:**

This study aimed to strengthen culturally safe evaluation practice in Indigenous settings by engaging evaluation stakeholders, in both countries, in a participatory concept mapping study. Concept maps for each country were generated from multi-dimensional scaling and hierarchical cluster analysis.

**Results:**

The 12-cluster Australia map identifies four cluster regions: An Evaluation Approach that Honours Community; Respect and Reciprocity; Core Heart of the Evaluation; and Cultural Integrity of the Evaluation. The 11-cluster New Zealand map identifies four cluster regions: Authentic Evaluation Practice; Building Māori Evaluation Expertise; Integrity in Māori Evaluation; and Putting Community First. Both maps highlight the importance of cultural integrity in evaluation. Differences include the distinctiveness of the ‘Respecting Language Protocols’ concept in the Australia map in contrast to language being embedded within the cluster of ‘Knowing Yourself as an Evaluator in a Māori Evaluation Context’ in the New Zealand map. Participant ratings highlight the importance of all clusters with some relatively more difficult to achieve, in practice. Notably, the ‘Funding Responsive to Community Needs and Priorities’ and ‘Translating Evaluation Findings to Benefit Community’ clusters were rated the least achievable, in Australia. The ‘Conduct of the Evaluation’ and the ‘Prioritising Māori Interests’ clusters were rated as least achievable in New Zealand. In both countries, clusters of strategies related to commissioning were deemed least achievable.

**Conclusions:**

The results suggest that the commissioning of evaluation is crucial as it sets the stage for whether evaluations: reflect Indigenous interests, are planned in ways that align with Indigenous ways of working and are translated to benefit Indigenous communities Identified strategies align with health promotion principles and relational accountability values of Indigenous approaches to research. These findings may be relevant to the commissioning and conduct of Indigenous health program evaluations in developed nations.

## Background

In recent decades, investments have been made in programs, services and initiatives to address inequities and improve Indigenous people’s health and wellbeing in Australia [1] as well as in New Zealand [2]. These investments relate to ‘closing the gap’ between Indigenous and non-Indigenous populations in life expectancy, education, employment, access to services, housing, and other social wellbeing outcomes [3].

In Australia, the Productivity Commission [4] is calling for ‘more and better’ program evaluations to adequately capture the breadth and depth of change occurring in response to investment. In New Zealand, both the evaluations and the commissioning of evaluations have been identified as requiring improvement if they are to better address the interests and health needs of Māori [5].

Western approaches to evaluation are poorly equipped to accommodate the worldviews of Indigenous peoples’ and unlikely to contribute to the development of health programs that make a positive difference for Indigenous communities. The limited number and quality of evaluations is problematic and is viewed, in the Australian context, as stemming from a lack of culturally safe practice that effectively engages Indigenous peoples [6–8]. In the New Zealand context, poor cultural ‘fit’, or congruency, between evaluator and evaluand, contributes to the ineffective evaluation and evaluation commissioning of programs that target Indigenous service users [9]. Culturally safe evaluations meet and address the needs of Indigenous people, organisations and communities from an Indigenous cultural worldview or standpoint [10]. They require evaluation stakeholders to be self-aware of their cultural biases and assumptions, and the power they exercise in their relationships with Indigenous people, organisations and communities in all aspects of the evaluation process [11].

Many government and not-for-profit evaluations are driven by top-down models of accountability built around outcomes defined by the funder with a focus on ‘value for money’ or ‘social return on investment’ [12]. Externally-defined outcomes rarely capture Indigenous peoples’ measures of program success and often align poorly with community needs and priorities, disempowering Indigenous peoples and undermining their right to self-determination.

Internationally, it is recognised that evaluations of Indigenous health programs must balance methodological quality with cultural appropriateness [13]. Health services that are culturally unsafe act as barriers to Indigenous participation [14]; so, too, are evaluations that do not align with Indigenous cultural protocols and build respectful relationships [15].

There are critical differences between Australia and New Zealand in terms of better positioning evaluation to meet the needs of Indigenous communities. In Australia, the shortage of Indigenous evaluators contributes to an ongoing demand for culturally safe non-Indigenous evaluators, who often lead the evaluation of Indigenous programs. Here, cultural safety may be a priority requiring non-Indigenous parties to learn about Indigenous beliefs and values and, through critical self-reflection, identify the personal biases and White privilege that they bring to their evaluation practice. Becoming culturally safe is a process of understanding and transformation that can occur at individual (e.g., individual evaluator) and collective levels (e.g., health agency). The evaluation of Indigenous health and wellbeing programs must privilege equity in power relationships.

In the New Zealand context, Kaupapa Māori evaluation knowledge and practice have a firm ‘stake in the ground’. Māori evaluators are well represented across the evaluation community, and it is widely accepted that Māori evaluators will lead program evaluations targeting Māori participants [16]. A cultural ‘fit’ between evaluator, program provider, and program user, is also advocated by some evaluation stakeholders. Cultural fit means being an ‘insider’ who is able to share in the lived experience, understandings, cultural values, characteristics and language of Indigenous stakeholders [9].

Roles for non-Māori evaluators in the evaluation of Māori-driven programs may include providing support, if invited to do so, in a form determined by Māori-led evaluation teams. In these circumstances, some level of cultural competency is expected, inclusive of understanding New Zealand’s colonial history and its consequences. In-depth understanding of one’s own cultural identity and the limitations that identity may present in the evaluation context is also important [17]. Engaging in ongoing cultural competency development is integral to the sound practice of non-Māori stakeholders in the New Zealand evaluation space.

Although the need for culturally competent and safe evaluation practice is recognised across a range of health and wellbeing programs in Australia and New Zealand [18, 19], many evaluation stakeholders are unsure about what they can do in practice to ensure that the evaluation of Indigenous programs is a safe space for Indigenous leaders, providers and communities. This research was driven by Indigenous and non-Indigenous evaluators in Australia and New Zealand who recognised the need to engage evaluation stakeholders in a first-time ground-up process to identify strategies to support culturally safe evaluation practice in health promotion and health services in their respective countries. To inform practical action, the objectives of this research were to:

1. Identify strategies and practical actions to support culturally safe evaluation.
2. Develop a concept map of strategies and practical actions to support culturally safe evaluation in each country, i.e., Australia and New Zealand.
3. Rate the strategies and practical actions in relation to their perceived importance and feasibility to implement.
4. Disseminate and translate the findings to support culturally safe evaluation.

## Methods

Participatory concept mapping methodology [20] was utilised to generate and cluster strategies that support culturally safe evaluation practice in Indigenous settings in Australia and New Zealand. This mixed-method program planning approach has evolved over nearly 35 years and has been widely used in public health, health promotion, and medicine [21, 22]. It provides a structured process for gaining, organising and prioritising stakeholder perspectives on a given topic. Concept mapping engages stakeholders in three activities: (1) brainstorming statements in response to a prompt question; (2) sorting a refined set of statements into conceptually meaningful piles and labelling the piles; and (3) rating statements on their perceived importance and achievability. Both qualitative and multivariate statistical techniques are utilised to represent stakeholders’ ideas visually in a series of interpretable two-dimensional maps [20]. The visual display of the data has appeal for knowledge translation and influencing policy and practice.

### Project advisory group

A Project Advisory Group (PAG) was formed, with representation from Australian and New Zealand-based organisations. The PAG had Indigenous co-chairs; one from the South Australian Department of Health and Wellbeing and the other from Whakauae Research for Māori Health and Development. The PAG met two to three times a year via teleconference to provide strategic advice on critical aspects of the research process.

### Data collection

To address the study objectives, evaluation stakeholders were engaged in brainstorming, sorting and rating activities. Before participating in these activities, stakeholders completed a set of demographic questions.

The study protocol was reviewed by the University of South Australia Human Research Ethics Committee, the Aboriginal Health Council of South Australia Human Research Ethics Committee, and the New Zealand Health & Disability Ethics Committee.

### Demographic questions

Participants indicated their:

- Primary and secondary roles supporting or practicing evaluation in Indigenous settings;
- Years of experience supporting or practicing evaluation in Indigenous settings and non-Indigenous settings;
- Country in which they primarily practiced or supported evaluation;
- Indigenous status; and
- Gender

Response categories are given in Table 1.

**Table 1:** Demographic characteristics of participants by concept mapping activity

Categories	Brainstorming^a^ (*n* = 70)		Sorting (*n* = 56)		Rating (*n* = 117)	
Country						
Australia	48	(68.5%)	30	(53.0%)	63	(53.9%)
New Zealand	20	(28.5%)	26	(47.0%)	54	(46.1%)
No response	2	(3.0%)	0	(0.0%)	0	(0.0%)
Gender						
Women	57	(81.4%)	45	(80.4%)	89	(76.1%)
Men	10	(14.3%)	10	(17.8%)	27	(23.0%)
No response	3	(4.3%)	1	(1.8%)	1	(0.9%)
Ethnicity						
Aboriginal/ TSI	16	(22.9%)	13	(23.2%)	25	(21.4%)
Māori	14	(20.0%)	14	(25.0%)	27	(23.1%)
Pasifika	1	(1.4%)	0	(0.0%)	3	(2.6%)
Non-Indigenous	37	(52.9%)	28	(50.0%)	62	(53.0%)
No response	2	(2.9%)	1	(1.8%)	0	(0.0%)
*Primary Role*						
External evaluator	29	(41.4%)	11	(28.6%)	42	(35.9%)
Policy, funder, admin	7	(10.0%)	7	(12.5%)	16	(13.7%)
Facilitator, coordinator	7	(10.0%)	10	(17.9%)	22	(18.8%)
Internal evaluation	7	(10.0%)	12	(12.5%)	14	(12.0%)
Community	1	(1.4%)	2	(3.6%)	6	(5.1%)
Capacity-building	6	(8.6%)	8	(14.3%)	7	(5.9%)
Other roles	6	(8.6%)	6	(10.7%)	0	(0.0%)
No response	7	(10.0%)	3	(5.4%)	10	(8.6%)
*Secondary Role*						
External evaluator	6	(2.3%)	2	(3.6%)	11	(9.4%)
Policy, funder, admin	4	(5.7%)	7	(12.5%)	9	(7.7%)
Facilitator, coordinator	4	(5.7%)	11	(19.6%)	17	(14.5%)
Internal evaluation	8	(11.4%)	3	(5.4%)	14	(12.0%)
Community	7	(10.0%)	11	(19.6%)	29	(24.8%)
Capacity-building	28	(40.0%)	14	(25.0%)	17	(14.5%)
Other roles	5	(7.1%)	5	(8.9%)	8	(6.8%)
No response	7	(10.0%)	3	(5.4%)	12	(10.3%)
Years’ Experience (Indigenous)						
< 2 years	13	(18.6%)	10	(17.9%)	25	(21.4%)
2–5 years	12	(17.4%)	13	(23.2%)	24	(20.5%)
5–10 years	10	(14.3%)	13	(23.2%)	25	(21.4%)
10–15 years	12	(17.1%)	6	(10.7%)	22	(18.8%)
15+ years	16	(22.9%)	13	(23.2%)	18	(15.4%)
No response	7	(10.0%)	1	(0.6%)	3	(2.6%)
Years’ Experience (non-Indigenous)						
< 2 years	14	(20.0%)	13	(23.2%)	32	(27.4%)
2–5 years	11	(15.7%)	7	(11.3%)	21	(18.0%)
5–10 years	16	(12.9%)	10	(17.9%)	15	(12.8%)
10–15 years	6	(8.6%)	11	(19.6%)	21	(18.0%)
15+ years	16	(22.9%)	14	(25.0%)	21	(20.5%)
No response	7	(10.0%)	1	(0.6%)	4	(3.4%)

^a^For the online brainstorming activity only. Does not include demographics for the 30 workshop participants

### Step 1: Brainstorming

The brainstorming activity was guided by the following focus prompt generated by the PAG: *To ensure the planning and conduct of an evaluation benefits Indigenous people, these are the things that I MUST think, feel, see and/or do.* Evaluation stakeholders responded to the prompt question through an online portal accessible through Concept Systems Global Max software. Invitations were emailed directly to 120 participants who had presented on Indigenous topics at the Australasian Evaluation Society (AES), Australian Health Promotion Association (AHPA) and/or Aotearoa New Zealand Evaluation Association (ANZEA) conferences and to the Australian Council for International Development’s Aboriginal and Torres Strait Islander Monitoring and Evaluation Learning Group.

The brainstorming activity took most participants about 10 min to complete. Also, approximately 30 evaluation stakeholders contributed by participating in small group workshops convened during AES and AHPA conferences in 2015. Online and face-to-face participation generated 350 and 30 statements, respectively. The face-to-face brainstorming did not generate any strategies different from those identified online.

The combined strategy statements generated across both countries were themed and consolidated, by eight members (four members from each country) of the research team. The theming process required the research team to navigate issues such as language variation (e.g., Indigenous, Aboriginal and Torres Strait Islander, Māori), use of voice (i.e. first person, second person, third person). The team resolved these issues in discussion with the PAG. Statements were consolidated from 350 to 106. The literature recommends condensing brainstorming results to no more than 100 statements [20]. However, the research team determined that any further reduction in the number of statements was neither acceptable nor achievable.

### Step 2: Sorting

The 106 consolidated statements were used in the face-to-face sorting activity. Following recommended procedures [20], participants individually grouped statements into conceptually meaningful piles labelling each using a word, or phrase, that for them best summarised the concepts included in that pile. Sorting workshops were held in Adelaide, Darwin, Melbourne, Cairns (Australia) and in Auckland, Whanganui and Wellington (New Zealand). Facilitators ensured procedural consistency within and between countries. Sorting packs were prepared with instructions and distributed to interested participants, who were unable to attend a workshop, to self-administer. Participants were recruited through the same mechanism used to recruit participants for the brainstorming activity as well as through direct email invitations sent to ANZEA members, using the membership contact list published on the ANZEA website, and to research team member networks. The sorting activity took, on average, 90 min to complete. Data collection occurred over 6 months and, on the advice of PAG, continued until the Indigenous participation rate in each country reached at least 40%.

### Step 3: Rating

Participants were invited to rate each of the 106 statements on perceived importance and perceived achievability. The following prompts guided assignment of the ratings:

- *How important* are each of the following practices or strategies in the design and delivery of evaluation in Indigenous settings; and
- *How achievable* it is to implement each of the following practices or strategies, in the design and delivery of evaluation in Indigenous settings, within the next 12 months.

Participants were asked to rate the statements, relative to each other, using a 5-point scale where 1 = not at all important/ not at all achievable and 5 = extremely important/ extremely achievable.

Participants were invited via email using the email lists used for recruitment in the brainstorming and/or sorting phases of the study. Ratings workshops were also held in Melbourne, Adelaide (Australia), Auckland and Whanganui (New Zealand).

### Data analysis

Sorting activity data was manually entered into Concept Systems Global Max software and separately analysed for each country in three steps.

A matrix of similarities was constructed showing the number of participants who sorted any pair of strategy statements in the same pile regardless of what other statements were sorted with them. This binary similarity matrix was the input for non-metric multidimensional scaling (MDS) analysis with a two-dimensional solution. MDS provided a two dimensional (x,y) configuration for the 106 statements based on the criterion that statements sorted together most often are located more closely in two-dimensional space than statements sorted together less frequently. The stress value from the point map indicates how well the data from the similarity matrix fit with the point map. Stress values typically range from 0.205 and 0.365 [20]; the lower the stress value, the closer the fit. The x,y configuration is the input for agglomerative hierarchical cluster analysis. There is no mathematical criterion to select the optimal cluster solution. A review of 69 concept mapping applications found a median of nine clusters and range of six to 14 clusters [22].

Research team members, in each country, started with a 14-cluster solution concept map and examined successively lower cluster solutions. Clusters were merged if conceptually or culturally reasonable and justifiable. Merging stopped when there was a conceptual or cultural basis for keeping clusters distinct. The analysis results in a ‘cluster map’ which displays the 106 statements enclosed by polygon-shaped distinct boundaries that indicate the clusters. Cluster labels were determined through an iterative process with evaluation stakeholders. Bridging values are associated with each cluster. Clusters with low bridging values indicate that statements within that cluster are firmly anchored to that part of the map. Clusters with high bridging values indicate that statements in that cluster were frequently sorted with distant statements, acting as a ‘bridge’ to other parts of the map.

For the rating data, average cluster ratings on perceived importance and achievability for each country were visually displayed using a “ladder” graph. Differences in cluster ratings for importance and achievability were assessed by Indigenous status. Average cluster ratings on importance and achievability, overall and by Indigenous status, were evaluated using independent t-tests.

## Results

### Participant characteristics

Participant demographic characteristics are summarised for the brainstorming, rating and sorting activities in Table 1.

Across the brainstorming, sorting and rating activities, there was greater representation from women and non-Indigenous participants, lesser representation of community members in a steering committee, or advisory, primary evaluation stakeholder role. There was greater input from Australian evaluation stakeholders in the brainstorming activity than from their New Zealand counterparts. A strong core of participants, across all activities, were highly experienced in evaluation in Indigenous and non-Indigenous settings.

Due to the anonymity and confidentiality of the data collection activities, we could not determine the proportion of participants completing multiple activities.

### Concept maps

On the advice of the PAG, concept maps were generated separately for each country to respect the cultural contexts of each country.

**Fig. 1:**
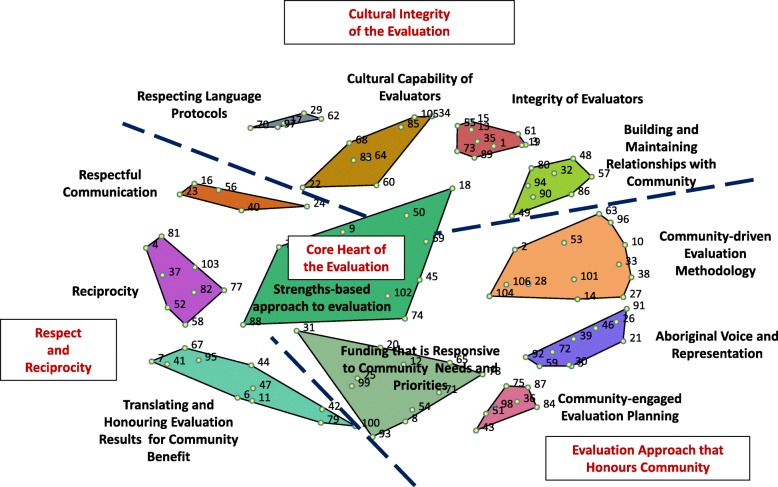
Concept map with 12 clusters in 4 regions (Australia)

For Australia, the stress value of our analysis with 30 sorters was 0.3528. A 12-cluster concept map was deemed the optimal solution by the Indigenous and non-Indigenous members of the research team from Australia. The Indigenous research team members believed that merging the ‘Language’ cluster with the ‘Integrity of Evaluators’ cluster would undermine the importance of language in an Indigenous Australian evaluation context where over 500 language groups exist. The merging of clusters was finalised at this stage of the analysis. Two groups of Indigenous and non-Indigenous sorters (*n* = 6) reviewed the 12-cluster solution, re-allocated a select number of points to adjacent clusters to enhance the conceptual clarity of the clusters, refined the cluster labels, and identified regional clusters on the map. Table 2 presents the three strategies for each of the 12 Australian clusters with the lowest bridging values. The final concept map, shown in Fig. 1, identifies the clusters according to the four regions of the map: (1) Cultural Integrity of the Evaluation; (2) Respect and Reciprocity; (3) An Evaluation Approach that Honours Indigenous Communities; and (4) Core Heart of the Evaluation. The two clusters in the ‘Respect and Reciprocity’ region (i.e., Respectful Communication and Reciprocity and Translation clusters) had the highest bridging values (0.85 and 0.77, respectively); items within these clusters were sorted with items in other parts of the map.

**Table 2:** Representative statements for each cluster in the Australia concept map with statement ID and bridging values

Cluster Statement			Bridging Value
1. Integrity of evaluators			
	13	Be humble, empathic, open, and honest.	0.00
	1	Observe with both eyes, listen with both ears and speak little.	0.02
	15	Talk the walk and walk the talk’ i.e., evaluators need to say what they are going to do, do what they say.	0.11
2. Building and maintaining relationships with community			
	32	Consider and address gender roles and responsibilities prior to starting the evaluation.	0.23
	49	Reflect on the connection of the evaluator/s with the community/iwi/hap? and the kaupapa or reason for the evaluation/project. How strong is the connection and how “good” is the fit? Is there someone else who should be here?	0.25
	80	Make time for evaluator/s and the community to “get to know each other”, make relationship connections and build trust early before the evaluation can move forward.	0.25
3. Community-driven evaluation methodology			
	106	The methods used to collect data are life affirming and meaningful for Indigenous evaluators and/or participants.	0.17
	104	The evaluation plan and approach build on the strengths of Indigenous people and culture.	0.21
	53	Capture the diversity of Indigenous peoples within and between communities.	0.23
4. Strengths-based approach to evaluation			
	45	Consider and address the power dynamics and relationships between the evaluators and participants.	0.20
	18	Ensure that integrity is at the forefront of the evaluation process.	0.22
	69	Conduct evaluation activities in a manner that enhances the standing of the Indigenous community including accommodating conflicting views and looking for ways forward.	0.23
5. Respecting language protocols			
	17	Correctly pronounce the Indigenous language of respective Indigenous communities.	0.38
	29	Pay attention to Indigenous people’s preference for language i.e., know when it is appropriate to use Indigenous language or not.	0.38
	62	Where necessary, ensure that an interpreter who is trusted and well regarded by the community is available.	0.38
6. Cultural capability of evaluators			
	34	Have a respected cultural advisor on the team.	0.20
	105	Non-Indigenous evaluators need to take responsibility and recognise the impact of their ‘whiteness’ including the increased opportunities this confers to exercise power and control.	0.24
	64	Those undertaking the evaluation have received cultural safety training, if non-Indigenous.	0.25
7. Reciprocity			
	103	Think about how to present unpalatable, difficult or challenging data - or even missing data - how can this be done so it doesn’t cause further harm?	0.71
	77	Include opportunities for Indigenous capacity building in the program and the evaluation.	0.71
	82	Train local Indigenous people to work on the evaluation.	0.80
8. Respectful communication			
	24	Ensure that the evaluator is able to inform the project and impact on the credibility of the evaluation findings and the integrity of all those involved.	0.58
	23	The language used to share evaluation information with the community is easy to follow so the community understands what is being done, and how they can be involved, if appropriate.	0.74
	56	Maintain confidentiality regardless of how minor the issue may be - keeping it confidential is critical.	0.77
9. Translating and honouring evaluation results for Aboriginal community benefit			
	7	Evaluation findings are adequately communicated to policy makers in the interests of effecting positive change.	0.38
	41	Consider and address how evaluation results may be translated into longer term benefits for the Indigenous community.	0.42
	95	Secure community endorsement for publication and reports	0.49
10. Aboriginal voice and representation			
	72	Engage community in planning and co-creation of the evaluation framework/model.	0.21
	92	Identify who the “community” is - ensure the community identified by the commissioner is actually the right community.	0.23
	5	Work with Indigenous people in the planning stages to find out what they want to know to ensure that the evaluation questions reflect their needs, issues and concerns.	0.28
11. Community-engaged evaluation planning			
	87	What Indigenous people value about the program/initiative is reflected in the evaluation questions and plan.	0.18
	43	That program objectives and targets have been defined by the community and not by an external party such as a funding body.	0.23
	84	Outcome measures are defined with the community to capture what is important to the community as well as the funding body.	0.28
12. Funding that is responsive to Aboriginal community needs and priorities			
	78	The evaluation terms of reference or activity plan is balanced so it meets the requirements/needs of the community and the agenda of the evaluation commissioner.	0.17
	71	Clearly and overtly define the power dynamics of all stakeholders and use this to assist in defining the purpose and audiences of the evaluation in the evaluation design.	0.30
	65	Ensure from the outset of planning that commissioners engage and consult with Indigenous people.	0.33

For New Zealand, the stress value of our analysis with 26 sorters was 0.3609. An 11-cluster concept map was deemed the optimal solution on the basis that: (a) the two clusters pertaining to ‘Relationship Building’ and ‘Participation’ (Clusters 12 and 11) comprising ‘Authentic Evaluation Methods’ were more meaningful when combined; and the clusters entitled (b) ‘Prioritising Māori Interests in Community’ (Cluster 9) and ‘Prioritising Community Interests in the Project and Evaluation Plan’ (Cluster 10) were distinct concepts from a Māori evaluation perspective. The New Zealand team went through the same process of ground-truthing the concept map by gaining input from a small group comprising two Indigenous and two non-Indigenous study participants.

**Fig. 2:**
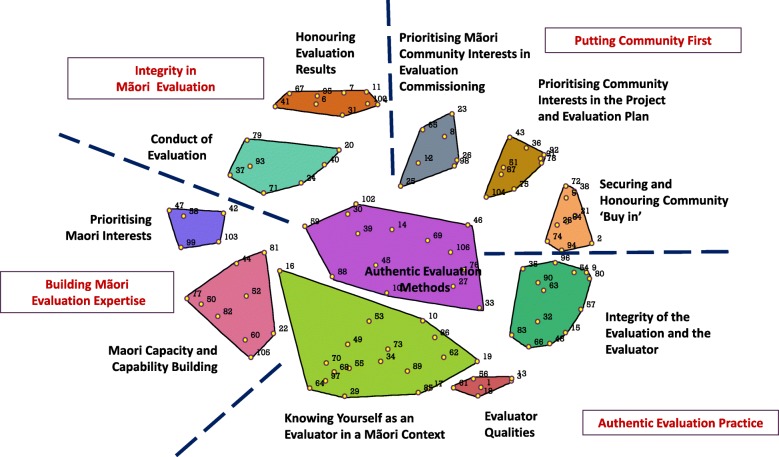
Concept map with 11 clusters in 4 regions (New Zealand)

The final concept map, shown in Fig. 2, identifies the clusters according to the four regions of the map: (1) Integrity in Māori Evaluation; (2) Putting Community First; (3) Building Māori Evaluation Expertise; and (4) Authentic Evaluation Practice. The two clusters in the Building Māori Evaluation Expertise region (‘Prioritising Māori Interests in Community’ and ‘Māori Capacity and Capability Building’) had the highest bridging values (0.79 and 0.65, respectively) suggesting that items in these clusters were sorted with items in other parts of the map. Table 3 presents three statements for each of the 11 New Zealand clusters (i.e., those with the lowest bridging values).

**Table 3.** Representative statements for each cluster in the New Zealand concept map with statement ID and bridging values

Cluster		Statement	Bridging Value
1. Evaluator qualities			
	1	Observe with both eyes, listen with both ears and speak little.	0.14
	13	Be humble, empathic, open, and honest.	0.14
	61	Have an open-mind in engaging or working with Indigenous people.	0.16
2. Knowing yourself as an evaluator in a Mãori context			
	10	Use culturally appropriate evaluation methods.	0.03
	17	Correctly pronounce the Indigenous language of respective Indigenous communities.	0.08
	89	Working in ways that are culturally appropriate.	0.08
3. Securing and honouring community ‘buy-in’			
	84	Outcome measures are defined with the community to capture what is important to the community as well as the funding body.	0.26
	72	Engage community in planning and co-creation of the evaluation framework/model.	0.28
	94	Negotiate and be flexible about timeframes in order to respect community priorities, events, and the changing availability and other responsibilities of key informants.	0.28
4. Integrity of the evaluation & the evaluator			
	35	Dialogue between the Indigenous community and evaluator/s needs to be prioritised in preference to one- way conversation.	0.20
	66	Build the principles of respect, reciprocity, and responsiveness into the evaluation.	0.20
	83	See evaluators taking the time to understand issues the Indigenous partners are facing, outside of the evaluation; it shows a respectful attitude towards the partners.	0.21
5. Prioritising Mãori community interests in evaluation commissioning			
	98	Establish an Indigenous governance structure so the evaluation project can be discussed at all stages with the community.	0.24
	26	Facilitate engagement with the Indigenous ‘owners’ of the evaluation and identify their values and worldviews against which to judge the evaluative data.	0.25
	65	Ensure from the outset of planning that commissioners engage and consult with Indigenous people.	0.28
6. Prioritising community interests in the project and evaluation plan			
	87	What Indigenous people value about the program/initiative is reflected in the evaluation questions and plan.	0.21
	104	The evaluation plan and approach build on the strengths of Indigenous people and culture.	0.21
	75	Consider and address whether scope is built into the evaluation to engage all stakeholders to ensure the evaluation benefits Indigenous people.	0.24
7. Authentic evaluation methods			
	106	The methods used to collect data are life affirming and meaningful for Indigenous evaluators and/or participants.	0.00
	46	Use measurement tools that have been developed by/for and validated within Indigenous populations.	0.05
	27	The evaluation approach must reflect an understanding of the Indigenous community/group’s history and context, issues, worldview and strengths, including the impact of colonisation.	0.05
8. Honouring evaluation results			
	67	Provide a publication space for Indigenous voices with Indigenous reviewers (culturally safe peer review).	0.12
	7	Evaluation findings are adequately communicated to policy makers in the interests of effecting positive change.	0.17
	6	A reflective process takes place post evaluation with Indigenous communities to enable key findings to be implemented to strengthen their work and achieve their goals.	0.27
9. Conduct of evaluation			
	20	Commissioners and providers of evaluation must ensure a good relationship is built with the Indigenous group (being evaluated) and that the Indigenous group is happy to proceed with the evaluation, and if not, they have other options/ evaluation teams provided to them.	0.33
	40	Commissioners of evaluation need to be mindful of how they interact with the Indigenous community; they need to communicate in ways that Indigenous communities feel comfortable responding to.	0.50
	79	Indigenous stakeholders own and control the intellectual property arising from the evaluation.	0.52
10. Prioritising Mãori interests			
	42	Must be Indigenous led or at the very least Indigenous people in positions of equal power as non-Indigenous people.	0.64
	47	No funding for evaluations should be given to organisations which do not employ Indigenous people in senior positions for the evaluation.	0.70
	58	Consult and negotiate monetary compensation with Indigenous people and organisations who have contributed to the evaluation.	0.79
11. Mãori capability and capacity building			
	22	Recognise and respect Indigenous evaluation capability.	0.53
	82	Train local Indigenous people to work on the evaluation.	0.56
	77	Include opportunities for Indigenous capacity building in the program and the evaluation.	0.61

### Rating

**Fig. 3:**
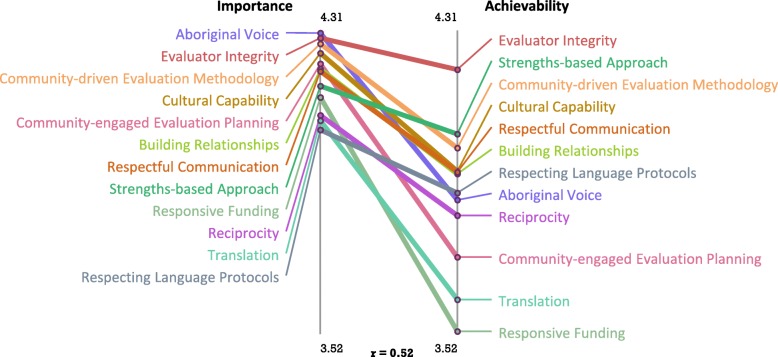
Pattern match of average perceived importance and achievability ratings (Australia)

The “ladder graph” or pattern match for Australia illustrates the level of agreement between average cluster ratings on perceived importance and achievability (Fig. 3). The rank order of the clusters on importance and achievability demonstrated a moderately strong correlation (r = 0.52). Table 4 shows the mean importance and achievability cluster ratings overall and by Indigenous status. Average cluster ratings for importance ranged from 4.07 to 4.31 (i.e., in the ‘very important’ range) and for achievability, from 3.52 to 4.22 (i.e., in the ‘moderately important’ to ‘very important’ range). The ‘Aboriginal Voice’, ‘Integrity of Evaluators, ‘Community-driven Evaluation Methodology’ and ‘Cultural Capability’ clusters had the highest average cluster ratings for importance. T-tests indicate that seven of the 12 clusters had higher average scores on importance than achievability (*p* < 0.05). Relative to other clusters, ‘Community-engaged Program Planning’, ‘Translation that Honours and Benefits Community’ and ‘Funding that is Responsive to Community Needs and Priorities’ were rated the lowest on achievability, but still in the ‘moderately achievable’ range.

**Table 4:** Mean importance and achievability ratings, standard deviation and sample size for overall clusters and clusters by Indigenous status (Australia)

	Overall	Overall	Non-IND	IND	Non-IND	IND
Cluster	IMP^a^	ACH^b^	IMP	IMP	ACH	ACH
Building Relationships	4.22 (0.54) 62	3.95 (0.62)^1^ 55	4.12 (0.57) 37	4.33 (0.48) 25	3.90 (0.68) 30	4.01 (0.54) 25
Evaluator Integrity	4.31 (0.59) 63	4.22 (0.67) 55	4.27 (0.61) 38	4.36 (0.56) 25	4.16 (0.73) 30	4.29 (0.61) 25
Community-driven eval methodology	4.29 (0.55) 63	4.01 (0.60)^1^ 55	4.23 (0.61) 38	4.36 (0.56 25	3.90 (0.61) 30	4.15 (0.57) 25
Strengths-based approach	4.18 (0.63) 63	4.05 (0.63) 55	4.12 (0.63) 38	4.26 (0.63) 25	3.97 (0.70) 30	4.16 (0.54) 25
Respecting Language	4.07 (0.71) 63	3.88 (0.64) 55	4.01 (0.74) 38	4.18 (0.67) 25	3.81 (0.64) 30	3.97 (0.64) 25
Cultural Capability	4.28 (0.59) 63	3.95 (0.70)^1^ 55	4.22 (0.62) 38	4.37 (0.53) 25	3.84 (0.79) 30	4.09 (0.57) 25
Reciprocity	4.11 (0.66) 63	3.82 (0.64) 55	4.02 (0.73) 38	4.25 (0.53) 25	3.74 (0.69) 30	3.92 (0.56) 25
Respectful Communication	4.21 (0.59) 63	3.93 (0.55) 55	4.17 (0.54) 38	4.26 (0.67) 25	3.98 (0.54) 30	3.89 (0.57) 25
Translation	4.09 (0.60) 63	3.61 (0.69)^1^ 55	3.98 (0.64) 38	4.28 (0.49) 25	3.43 (0.72)^1^ 30	3.85 (0.58)^1^ 25
Aboriginal Voice	4.32 (0.50) 63	3.87 (0.66)^1^ 55	4.30 (0.49) 38	4.35 (0.52) 25	3.75 (0.67) 30	4.00 (0.65) 25
Community-engaged plan	4.23 (0.59) 62	3.72 (0.73)^1^ 54	4.12 (0.65) 37	4.40 (0.46) 25	3.58 (0.76) 29	3.88 (0.68) 25
Responsive Funding	4.14 (0.59) 63	3.52 (0.77)^1^ 55	4.08 (0.58) 38	4.23 (0.61) 25	3.30 (0.77)^1^ 30	3.77 (0.70)^1^ 25

^1^ p < 0.05 ^a^ IMP = Importance ^b^ ACH = Achievability

Importance ratings did not differ by Indigenous status. The ‘Translation’ and ‘Responsive Funding’ clusters were assigned lower ratings on achievability by non-Indigenous stakeholders than they were by Indigenous stakeholders.

**Fig. 4:**
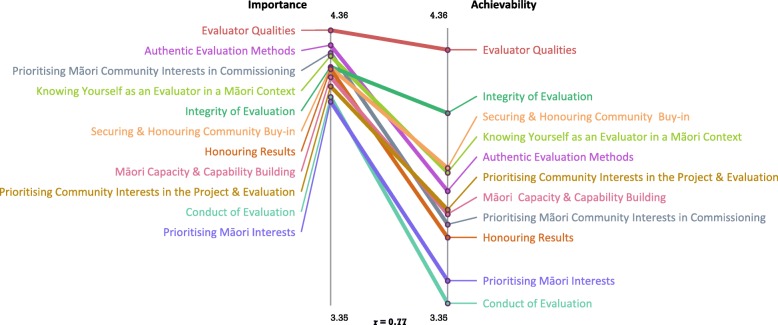
Pattern match of average perceived importance and achievability ratings (New Zealand)

The ladder graph for New Zealand illustrates the level of agreement between average cluster ratings on perceived importance and achievability (Fig. 4). The correlation between relative importance and achievability at the cluster level was strong (*r* = 0.77). Table 5 shows the mean importance and achievability cluster ratings overall and by Indigenous status. Average cluster ratings ranged from 4.14 to 4.47 for importance and from 3.35 to 4.36 for achievability. T-tests indicate that nine of the 11 clusters had statistically significant higher average ratings on importance than achievability. ‘Evaluator Qualities and ‘Integrity of the Evaluation’ were the only clusters with similar ratings on importance and achievability.

**Table 5:** Mean importance and achievability ratings, standard deviation and sample size for overall clusters and clusters by Indigenous status (New Zealand)

	Overall	Overall	Non-IND	IND	Non-IND	IND
Cluster	IMP^a^	ACH^b^	IMP	IMP	ACH	ACH
Evaluator qualities	4.47 (0.64) 54	4.36 (0.66) 44	4.41 (0.78) 27	4.52 (0.46) 27	4.36 (0.49) 19	4.37 (0.77) 25
Knowing yourself as an evaluator	4.33 (0.53) 53	3.87 (0.75)^1^ 44	4.17 (0.59) 26	4.49 (0.43)^1^ 27	3.75 (0.37) 19	3.96 (0.94) 25
Securing buy-in	4.31 (0.56) 54	3.89 (0.78)^1^ 44	4.43 (0.45) 27	4.47 (0.47)^1^ 27	3.71 (0.51) 19	4.01 (0.93) 25
Integrity of the evaluation	4.29 (0.55) 53	4.11 (0.69) 44	4.12 (0.63) 26	4.45 (0.41)^1^ 27	4.00 (0.44) 19	4.19 (0.84) 25
Prioritising Maori interests in commissioning	4.36 (0.52) 54	3.67 (0.82)^1^ 44	4.26 (0.56) 27	4.45 (0.47) 27	3.49 (0.65) 19	3.80 (0.92) 25
Prioritising community interests	4.22 (0.61) 51	3.72 (0.78)^1^ 44	4.02 (0.69) 24	4.39 (0.48)^1^ 27	3.51 (0.54) 19	3.88 (0.90) 25
Authentic evaluation methods	4.38 (0.46) 53	3.80 (0.76)^1^ 44	4.28 (0.52) 26	4.47 (0.37) 27	3.69 (0.47) 19	3.88 (0.93) 25
Honouring results	4.30 (0.57) 54	3.61 (0.78)^1^ 44	4.14 (0.64) 27	4.46 (0.44)^1^ 27	3.47 (0.61) 19	3.72 (0.89) 25
Conduct of evaluation	4.17 (0.64) 52	3.35 (0.94)^1^ 44	3.99 (0.70) 25	4.35 (0.53)^1^ 27	3.05 (0.78) 19	3.58 (1.01) 25
Prioritising Maori Interests	4.14 (0.56) 51	3.44 (0.87)^1^ 44	3.99 (0.50) 24	4.29 (0.59) 27	3.22 (0.68) 19	3.61 (0.98) 25
Maori capacity & capability	4.25 (0.50) 52	3.71 (0.80)^1^ 44	4.05 (0.49) 25	4.43 (0.45)^1^ 27	3.54 (0.55) 19	3.83 (0.93) 25

^1^ *p* < 0.05 ^a^ IMP = Importance ^b^ ACH = Achievability

Average cluster ratings on importance and achievability were assessed by Indigenous status. Average cluster ratings on importance were higher among Indigenous participants for seven clusters: ‘Knowing Yourself as an Evaluator’, ‘Securing Buy-in’, ‘Integrity of the Evaluation’, ‘Prioritising Community Interests’, ‘Honouring Results’, ‘Conduct of the Evaluation’ and ‘Māori Capability’. For achievability, there were no statistically significant differences in average cluster ratings.

### Dissemination and translation

Research findings were disseminated to national conferences in each country in addition to the Department of Prime Minister and Cabinet in Australia. Cultural guides are under development to strengthen evaluation practice. The results led to a successful grant submission focused on identifying strategies to strengthen Indigenous leadership and engagement in the commissioning of Indigenous health and wellbeing program evaluations in Australia.

## Discussion

This study responds to a need to identify strategies and practical actions to support culturally safe evaluation in Indigenous settings in Australia and New Zealand. Although cultural safety has been long-identified as an evaluation principle [23, 24], no empirical studies with broad-based Indigenous and non-Indigenous evaluation stakeholder consultation have explicitly identified strategies to support this principle. This study is novel for taking a ground-up participatory approach across two countries to strengthen the evidence base on the practice of culturally safe evaluation. Our findings align with evaluation practitioners’ time-honoured reflections and insights in New Zealand [25] and Australia [26]. These findings additionally align with health promotion principles which are underpinned by an ethos of social justice and with processes that foster empowerment and self-determination to reduce health disparities [27]. It is anticipated that improving the cultural safety of evaluations will strengthen the evidence base on what programs are effective in ‘closing the gap’ and why. Along with their similarities, the strategies reflect differences in the social and cultural contexts of Australia and New Zealand. Many of these differences are historically situated, reflecting the specific processes and experience of colonisation in each country. The ratings that study participants assigned to the strategies highlight that the ‘Integrity of Evaluators’ and ‘Evaluator Qualities’ clusters are both important and most achievable relative to other clusters in Australia as well as in New Zealand. Specific statements for these clusters can be viewed in Table 2 (cluster 1) and Table 3 (cluster 1).

### New Zealand

The 11-cluster concept map for New Zealand is influenced by a unique history of some four decades of Māori reclamation of the research and evaluation space; the growing number of Māori evaluators; and the more recent evolution of evaluation leadership, through Mā te Rae, Māori Evaluation Association formed in 2015 and through ANZEA, that prioritises cultural integrity. Cultural integrity is recognised as being pivotal to evaluation that ‘works’ for Māori. Cultural integrity includes the foundational expectation that evaluators and commissioners will be aware of their own cultural identity; who they are, where they are from, where they ‘fit’ in a contemporary New Zealand society shaped by colonisation and what the implications of their cultural identity are for their practice. Though woven throughout the New Zealand concept map, cultural integrity features prominently in the ‘Authentic Evaluation Practice’ cluster region and within its two largest, and central clusters; ‘Knowing Yourself as an Evaluator in a Māori Context’ and ‘Authentic Evaluation Methods’ (see statements in Table 3, clusters 2, 7). Both clusters speak to evaluator reflexivity and to prioritising evaluation approaches that place Māori self-determination at their core. These findings are supported by a Māori “community-up” approach where evaluators “Respect people”, “Meet people face-to-face”, “Look and listen”, “Share, host and be generous”, “Be cautious”. “Do not trample on the dignity or a person” and “Be humble” [25].

### Australia

The positioning of concepts in the 12-cluster concept map provides insights into evaluation practice to benefit Indigenous people in Australia. The ‘Aboriginal Voice and Representation’ cluster borders both the ‘Community-driven Evaluation Methodology’ and ‘Community-engaged Evaluation Planning’ clusters highlighting the significance of Aboriginal cultural guidance to both evaluation planning and methodology. The proximity of ‘Responsive Funding’ suggests that commissioners need to work with Indigenous communities to ensure programs fit with community needs and priorities, and similarly that the evaluation questions, plan and outcomes reflect what Indigenous people value. This finding is supported by a recent Indigenous Australian wellbeing framework which highlights a ‘shared space’ approach where government (e.g., commissioners), non-government (e.g., evaluators) and Indigenous community stakeholders work collaboratively [28]. For evaluation, the implication is that all evaluation decision-making from generating the evaluation questions to the translation of evaluation findings are conducted within this ‘shared space’. Brainstormed community planning strategies support an integrated approach to planning and evaluation, such that an evaluation plan aligns with a program’s objectives, a principle supported by recent Indigenous evaluation frameworks [29] and recommendations [30]. The upfront integration of program planning with evaluation, at the commissioning stage, through the collective decision-making of evaluation stakeholders in a shared space [28] is reflective of ‘co-design’, an emerging model in Indigenous evaluation practice in Australia [31] and the self-determination driver of participatory approaches to research and evaluation, more generally [32].

An evaluation’s cultural integrity must consider the cultural capability of the evaluation team and include formal roles for Indigenous community members, and evaluators. Having Indigenous expertise on the team enhances non-Indigenous evaluator capabilities to honour cultural ways of knowing and doing, including demonstrating respect for culture. There is an explicit link between Indigenous culture, worldview and traditional language which makes the presence of local Indigenous expertise essential for navigating and negotiating the evaluation with Indigenous communities [33]. With over 500 Aboriginal language groups in Australia, it is not expected that non-Aboriginal evaluators will be literate in local languages; instead, it is expected that evaluators will work with cultural advisors to ensure that local language is drawn upon in culturally appropriate ways like, for example, helping evaluators understand how language is central to their worldview and the program story (see statements in Table 2, cluster 5). A recent scoping review of the grey literature in Indigenous evaluation in Australia, New Zealand, Canada and the U.S. did not identify language as an evaluation principle or concept [23]. This finding contrasts with our study, which involved ground-up consultation, across two countries, and resulted in the engagement of many evaluation stakeholders with 10+ years of Indigenous evaluation experience (i.e. minimum 33% of participants in each activity). The voices of Indigenous evaluation stakeholders, in both countries, signal that Indigenous language matters and further, that future gains in Indigenous evaluation practice require face-to-face consultation [25]. This empirical finding from Australia may apply to First Nation populations in Canada, and Native Americans as these countries share diversity in traditional languages.

### Comparing the maps

Both the Australian and New Zealand maps highlight the importance of cultural integrity in evaluation though how that has been translated reflects each country’s own unique history of colonisation and its aftermath. In Australia, ‘Cultural Integrity of the Evaluation’ is reflected in the capacity of evaluation stakeholders to look inward, act in ways that respect culture and language, and build relationships with Indigenous communities (see statements in Table 2, Clusters 1,2,5,6). Cultural integrity is highlighted in the National Health and Medical Research guidelines for working with Indigenous Australian populations [34], which extends to evaluation. For New Zealand, ‘Integrity in Māori Evaluation’ is linked more firmly to interactions with commissioners (Conduct of Evaluation, Table 3 Cluster 9) and evaluators working with community to interpret and translate the results to influence Māori health directly or indirectly through policy. This empirical finding, resonates with the understandings that relationships in evaluation are pivotal, and that evaluator accountabilities to participants extend beyond the life of an evaluation [35]. In both maps, strategies related to the commissioning of evaluation (i.e., ‘Conduct of Evaluation’ (New Zealand) and ‘Responsive Funding’ (Australia) are similarly adjacent to translating the evaluation results to honour and benefit community. To our knowledge, this is a new finding and contribution to Indigenous evaluation.

Differences between the maps include language being firmly embedded within the cluster of ‘Knowing Yourself as an Evaluator in a Māori Evaluation Context’ in the New Zealand map in contrast to the distinctiveness of a ‘Respecting Language Protocols’ cluster in the Australian map. In the New Zealand context, the centrality of Te Reo Māori to Māori cultural identity is reflected in its integration within the ‘Knowing Yourself as an Evaluator in a Māori Evaluation Context’ cluster. The strong language theme across the cluster reinforces the recognition that at least some familiarity with, or commitment to developing familiarity with, Te Reo Māori is integral to the practice of all evaluators in New Zealand (see statements in Table 3, Cluster 2).

The concept maps reaffirm the importance of relational accountability in the conduct of evaluation in Indigenous settings, one where evaluation stakeholders (e.g., commissioners, evaluation consultants, service providers) plan and evaluate programs in ways that strengthen Indigenous peoples’ cultural identity, capability and wellbeing [36]. An Indigenous world is relationship-based; evaluations, in their design and implementation, need to respect Indigenous peoples’ relationships with others, the environment and the spirit world [10, 37]. Relational accountability is reflected throughout the concept maps as exemplified, for example, in the ‘Building and Maintaining Relationships with Community’, ‘Respectful Communication’, ‘Respecting Language Protocols’ and ‘Reciprocity’ clusters for Australia and in the ‘Securing and Honouring Community Buy-in’, ‘Prioritising Maori Community Interests in Commissioning’ and ‘Integrity of the Evaluation and the Evaluator’ clusters for New Zealand. Evaluation stakeholders must listen, acknowledge and create spaces for the perspectives and knowledge systems of Indigenous leaders and community members at the program and evaluation design stages. This includes Indigenous stakeholders having a say in defining program and evaluation outcomes. From an Indigenous perspective, all evaluation involves appropriation; evaluation must, therefore, be conducted for community benefit. As illustrated in both concept maps, evaluation results must be ‘honoured’ and ‘translated’ which may involve securing community endorsement for publication and reports. Evaluation must also respect Indigenous peoples’ rights and facilitate their ownership of the evaluation process and of the knowledge generated [36] like, for example, in establishing governance structures so evaluation projects can be discussed at all stages with community. Relational accountability is the cornerstone of Indigenous epistemology; it is therefore critical that evaluation stakeholders build respectful relationships with Indigenous communities in synchronicity with their knowledge systems and values to generate meaningful knowledge on how programs work in Indigenous settings [8, 25].

Relationships are also central to health promotion policy and practice their significance supported by the Ottawa Charter for Health Promotion’s remit to ‘enable people to increase control over and to improve their health’ (World Health Organization 1986). Translated to the New Zealand context, evaluation processes must support evaluation by, with and for Māori. In Australia, where Indigenous evaluation capacity is emergent, evaluation of any health, education and wellbeing programs must support participatory processes and leadership by Indigenous peoples. Study findings may be informative for evaluation stakeholders working with Indigenous populations in other developed nations as Indigenous populations share an epistemology of relational accountability and a holistic approach to health [36]. Globally, participatory approach is preferred to evaluating programs with Indigenous populations, as it supports self-determination [32] and community ownership, the latter of which has been linked to program success [38].

### Influence of context

Despite similarities in the two concept maps, the contexts within which evaluations are conducted within each country, differ. Compared to Australia, New Zealand has proportionately more Indigenous evaluators; evaluators who have collectively formed their own national evaluation organisation, Mā Te Rae to support and advance the interests of iwi Māori in the evaluation space. In Kaupapa Māori theory, evaluators have a well-established theoretical basis and evaluation epistemology to anchor their approaches to evaluation. Kaupapa Māori theory is internationally recognised [24] and has been used extensively to guide evaluations of health programs and services [39, 40]. Māori evaluators are taking responsibility for ensuring that evaluations are culturally sound and holding mainstream organisations accountable. The state of evaluation in New Zealand may reflect the acknowledgement of Indigenous rights with a treaty embedded in legislation obligating the government to a duty and commitment to partnerships with Indigenous communities.

When presenting the study findings to conference participants in each country, the research team noted differences in responses between the countries. Whilst in New Zealand, the predominant reaction from conference participants was “So what? We already know this. We’re doing it” in Australia, the response was much more akin to “This is new”.

These contextual differences may partially explain the higher average importance ratings assigned by New Zealand participants compared to those assigned by Australian participants. The lower achievability ratings in both countries likely reflect the perceived difficulty of enacting these strategies, particularly those strategies related to evaluation commissioning. Evaluators often operate within the restricted timeframes of short-term government funding cycles (e.g., 3 to 4 years) which may be insufficient for demonstrating effectiveness particularly when decisions need to be made collectively (in a ‘shared space’) and the evaluator must respect cultural protocols and community timeframes [41]. This type of contracting environment has been characterised as, ‘formalised, prescriptive and predicated on compliance’ [42] (pg 63). It has also been noted that government and Indigenous community stakeholders value success differently. Whereas government may evaluate success in terms of cost-effectiveness the community may be guided by their worldview in defining program success [31] [5]. Typically, government commissioning is supported by higher-level policies with performance measures and frameworks aimed at improving Indigenous health outcomes. That strategies related to evaluation commissioning (i.e., ‘Conduct of Evaluation’ (New Zealand) and ‘Funding that is Responsive’ (Australia)) are adjacent to honouring/ translating evaluation results highlights the importance of commissioners establishing good working relationships with Indigenous stakeholders at the beginning of the evaluation process (see statements in Table 3 clusters 8,9 and Table 2, clusters 9,12, respectively). This allows for evaluation results to be returned to, discussed with and actioned with or by the community. In practice, evaluators working with Indigenous communities do not always have control over disseminating evaluation findings [41]. In some tendering processes evaluators are contractually obligated to return the results to commissioners, and the commissioners own the intellectual property. In some instances, Indigenous communities are unaware of the results, despite providing resources to support the evaluation.

## Strengths and limitations

This study should be interpreted within the context of its strengths and limitations. A strength was the comprehensive process of evaluation stakeholder engagement, over an extended period, straddling two countries and including three cumulative data collection phases; brainstorming; sorting, and rating. Engagement was initiated when members of the research team presented study aims at two key conferences in 2014; AES and Equity @ the Centre: Action on the Social Determinants of Health. Ongoing engagement and data collection occurred, through until the end of 2017. In the spirit of relational accountability, this study aimed to be ground-up and participatory. Limitations include not being able to conclusively link participant data across the brainstorming, rating and sorting activities. We are aware, however, that some participants contributed to two or more phases of data collection. Follow-up interviews would have provided insight into difference in ratings between Indigenous and non-Indigenous participants. Additionally, the demographic characteristics of the 30 workshop participants contributing to the brainstorming were not collected. They are reported in the data collection step to reflect the breadth of input and consultation by the project.

## Conclusions

This study identified strategies and practices to support culturally safe evaluation in Indigenous settings in Australia and New Zealand. The concept maps for each country depict similarities as well as some differences. Participatory evaluation approaches that are led by and engage Indigenous peoples contribute to culturally safe evaluations. Needed are formal governance structures and processes that include Indigenous representation and leadership in evaluation decision-making. Sound evaluation planning and translation require evaluators and Indigenous stakeholders to establish and maintain good communication and working relationships with each other and with evaluation commissioners in the health system. The development of these relationships can be supported by more realistic (i.e., longer) timeframes to evaluate programs. Overall, in both countries, concepts supporting culturally safe evaluation had higher mean importance ratings than achievability ratings. A notable exception is the concept reflecting evaluator characteristics which was uniformly identified as most important and most achievable in both countries. Addressing evaluator characteristics represents ‘low-hanging fruit’ for professional associations and health agencies to action in the short-term by implementing cultural safety training workshops and establishing mentoring mechanisms. Although aligning evaluation commissioning with Indigenous interests is crucial to culturally safe evaluation practice it was identified as most challenging to action. Changing government systems will likely require a sustained long-term effort supported by a comprehensive Indigenous evaluation strategy. Evaluations that build-in Indigenous representation from the commissioning stage may, in the long-term, support government health policies that aim to ‘close the gap’ in health disparities in Australia and New Zealand as well as in other developed nations.
